# Early diagnosis of Gorlin-Goltz syndrome: case report

**DOI:** 10.1186/1746-160X-7-2

**Published:** 2011-01-25

**Authors:** Ana R Casaroto, Daniela CN Rocha Loures, Eduardo Moreschi, Vanessa C Veltrini, Cleverson L Trento, Vilmar D Gottardo, Vanessa S Lara

**Affiliations:** 1 Department of Oral Pathology, Bauru School of Dentistry, University of São Paulo, Bauru, Brazil; 2 Department of Dentistry, University Center of Maringá, Maringá, Brazil; 3 Department of Dentistry, Federal University of Sergipe, Aracaju, Brazil

## Abstract

The Gorlin-Goltz syndrome, also known as nevoid basal cell carcinoma syndrome (NBCCS), is an infrequent multisystemic disease inherited in a dominant autosomal way, which shows a high level of penetrance and variable expressiveness. It is characterized by keratocystic odontogenic tumors (KCOT) in the jaw, multiple basal cell nevi carcinomas and skeletal abnormities. This syndrome may be diagnosed early by a dentist by routine radiographic exams in the first decade of life, since the KCOTs are usually one of the first manifestations of the syndrome. This article paper reports the case of a patient, a 10-year-old boy with NBCCS, emphasizing its clinical and radiographic manifestations. This study highlights the importance of health professionals in the early diagnosis of NBCCS and in a preventive multidisciplinary approach to provide a better prognosis for the patient.

## Introduction

Nevoid basal cell carcinoma syndrome (NBCCS), also known as Gorlin-Goltz syndrome, is an autosomal dominant disorder characterized by a predisposition to neoplasms and other developmental abnormalities [1]. Gorlin & Goltz [2] described the classical triad composed of multiple basal cell carcinoma, keratocystic odontogenic tumors (KCOTs) in the jaws and bifid ribs that characterized the diagnosis of this syndrome. In addition to this triad, calcification of the falx cerebri, palmar and plantar epidermal pits, spine and rib anomalies, relative macrocephaly, facial milia, frontal bossing, ocular malformation, medulloblastomas, cleft lip and/or palate, and developmental malformations were also established as features of the syndrome [1,3].

This syndrome existed during Dynastic Egyptian times, as shown by findings compatible with the syndrome in mummies dating back to 1,000 b.c. [4]. The prevalence of NBCCS has been estimated from 1 in 57,000 [5] to 1 in 164,000 [6], but there is now general agreement that the prevalence is about 1 per 60,000 [7]. This syndrome probably presents itself in all ethnic groups, although a few cases have been published in certain human races, and affects both men and women in the same way [8].

During the last few years very important advances have taken place in the knowledge about the genetic characteristics of this syndrome [8]. The tumor suppressor gene called *Patched *(PTCH), located in the 9q22.3 chromosome, has been identified as cause of NBCCS [7,9]. However, mutations in others genes such as *Patched 2 *(PTCH2), *Smmothened *(SMO) and *Sonic hedgehog *(SHH) have been reported in isolated cases of basal cell carcinoma and medulloblastoma [3].

In the case of NBCCS it is of great importance to make an early diagnosis since the severity of complications, such as malignant skin and brain tumors can be reduced, and maxillofacial deformities related to the jaw cysts can be avoided [8]. The treatment of NBCCS involves a therapeutic approach to its clinical findings. The present report describes a patient with some typical features of NBCCS, which were diagnosed for the first time by preliminary orthodontic radiographic exams. Furthermore, the case emphasizes the importance of the dentist in recognizing these features in order to offer early diagnosis and a multidisciplinary approach to treatment of the syndromic patient.

## Case report

The patient, a 10 year-old white boy was the first child of non-consanguineous parents of normal stature (father's height, 180 cm; mother's height 165 cm). At the time of patient's birth, the father was 28 years old and the mother 25. The patient was born at 40 weeks of gestation after an uncomplicated pregnancy. In the present case, the syndrome did not affect the patient's parents and there were no familial antecedents.

**Figure 1:**
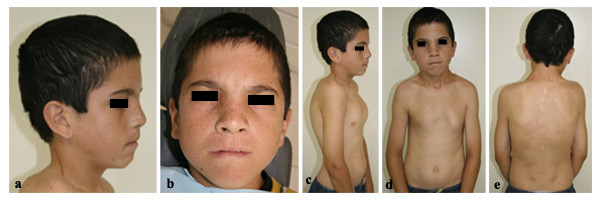
**Clinical features of NBCCS**. (a and b) Facial appearance of patient showed dysmorphic facial features, including relative macrocephaly (a) and ocular hypertelorism (b). (c and d) Lateral and frontal view showing pectum excavatum. (e) Vertebral anomaly characterized by cyphoscoliosis.

**Figure 2:**
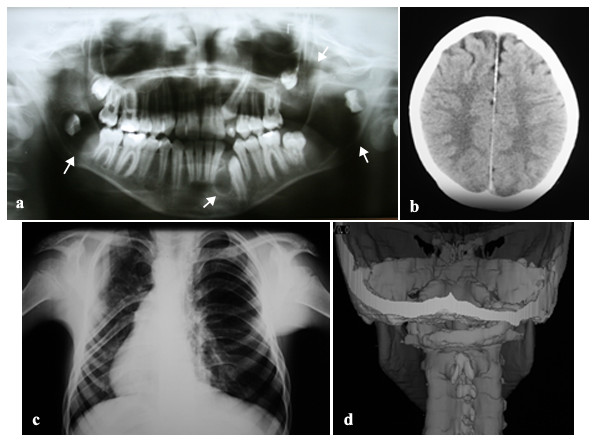
**Imaging findings of NBCCS**. (a) Orthopantographic examination suggesting the presence of multiple KCOTs in the maxilla and mandible (arrow). (b) Tomographic showing calcification of the cerebral falx. (c) Thorax film showing anomalies of the ribs characterized by flattening. (d) 3D tomographic reconstruction, showing spina bifida.

Initially, panoramic radiography for orthodontic purposes showed radiolucid images suggestive of multiple KCOTs in the jaws. Patient was then examined by an oral and maxillofacial surgery team for removal of the tumors. In the physical examination, however, the presence of dysmorphic facial features was observed, including relative macrocephaly (figure. 1a) and ocular hypertelorism (figure. 1b); pectum excavatum (figure. 1c and 1d), vertebral anomaly characterized by cyphoscoliosis (figure. 1e) and polydactyly of both hands. Other examinations were also performed which included, postero-anterior radiography of the skull and jaw, chest radiographs and computed tomography. In addition to images suggestive of KCOTs in maxilla and mandible (figure. 2a), imaging examinations revealed calcification of the falx cerebri (figure. 2b), rib anomalies (figure. 2c) and spine bifida (figure. 2d).

**Figure 3:**
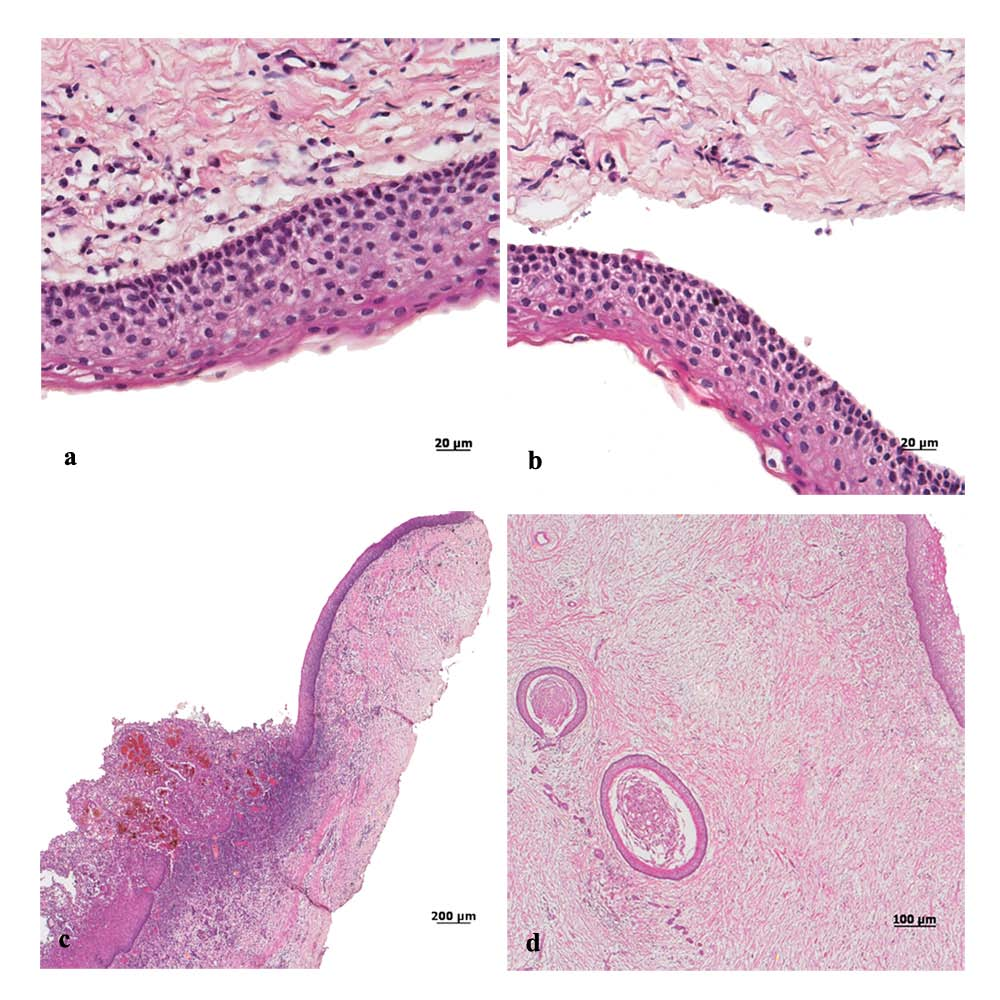
**Histopathology findings of KCOT**. (a and b) Prominent palisade basal cell layer with dark-staining nuclei and a corrugated surface with parakeratinization (H-E staining, original magnification × 40). (c) Presence of hyaline bodies and inflammatory changes that have destroyed parts of the lining ephitelium (H-E staining, original magnification × 4). (d) Prominent daughter cysts containing keratin whorls (H-E staining, original magnification × 10).

The tumors were surgically removed in consecutive sessions, through the enucleation and marsupialization technique. The specimens were fixed in 10% formalin and submitted to histopathological examination. The microscopic analysis showed epithelium with palisade basal cell layer with dark-staining nuclei and a corrugated surface with parakeratinization (figure. 3a and 3b). Prominent daughter-cysts containing keratin whorls were found in the thin capsular connective tissue (figure. 3d). In addition, tumors presented inflammatory changes, with consequent partial loss of epithelium lining features (figure. 3c). Based on clinical, radiographic and microscopic data, the hypothesis of KCOTs was confirmed and the diagnosis of NBCCS was established. The patient was referred to a dermatologist for appropriate dermatological care including investigation and early diagnosis of future skin lesions (basocellular carcinomas).

**Figure 4:**
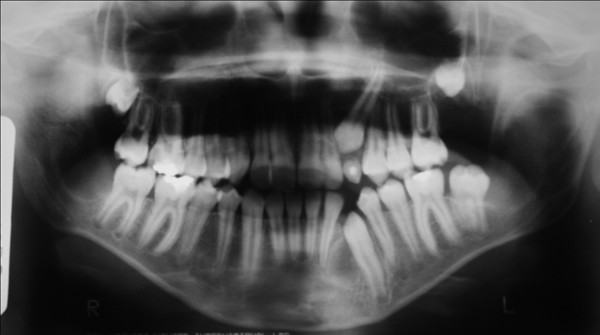
**Orthopantographic examination profile at three months follow-up after the surgery to remove the cystic lesions**.

New bone formation sites were identified in the three-month radiological follow-up (figure. 4). The patient and his parents are aware of the importance of regular examination.

## Discussion

Several studies have presented KCOTs, basal cell naevi and skeletal anomalies as the principal clinical features of NBCCS [3,10,11]. However, according to Manfredi et al. [10], the diagnostic criteria of NBCCS requires the presence of two major, or one major and two minor criteria. Major criteria included the presence of more than two basal cell carcinomas or one under the age of 20 years, histologically-proven KCOT of the jaw, cutaneous palmar or plantar pits, and bifid, fused or markedly splayed ribs. Any one of the following features is considered a minor criterion, such as orofacial congenital malformations (cleft lip or palate, frontal bossing or moderate or severe hypertelorism), skeletal and radiological abnormalities (bridging of the sella turcica and vertebral anomalies), ovarian fibroma and medulloblastoma.

The present case report showed a child patient presenting, among others, some of these features, such as multiple KCOTs in the maxilla and mandible, rib anomalies, spine bifida, calcification of the falx cerebri, ocular hypertelorism and vertebral anomaly characterized by kyphoscoliosis, which confirmed the diagnosis of NBCCS or Gorlin-Goltz syndrome.

One of the features found in this syndrome and emphatically mentioned in literature is the development of multiple basal cell carcinomas, especially in the head and neck region [1]. In this case it has not been possible to identify the presence of basal cell carcinomas. This fact can be explained by the patient's age (ten years old). Possibly, these carcinomas may develop in the future (second and third decade of life).

NBCCS is caused by mutations in a tumor suppressor gene PTCH (human homologue of a *Drosophila *segment polarity gene Ptch) located in chromosome 9q22.3 [1,3,12]. This protein can be found in the *Hedgehog *signaling pathway [8]. PTCH in the absence of its ligand, it acts as a cell-cycle regulator, normally inhibiting expression of downstream genes that control cell fate, patterning and growth [11]. Generally, for a tumor suppressor gene to be inactivated, two mutagenic hits (two distinct episodes of DNA damage) are required. The first hit involves a mutation in one allele, which can be dominantly inherited if present in a germ cell, but which is classically considered to have no phenotypic effect. The second hit involves loss of the other allele, known as loss of heterozygosity. When both alleles are inactivated, tumor growth occurs [3]. Loss of heterozygosity has been demonstrated in basal cell carcinomas, KCOTs and medulloblastoma, three features of NBCCS [3,8]. Various physical anomalies of the brain, ribs, vertebrae and limbs apparently need only one hit [11]. The single germ cell hit may account for the malformations and their variability in NBCCS patients [3].

According to Marotto et al. [13], some of the most common clinical findings of the syndrome are discovered through radiography commonly used in orthodontic treatment. In the case described in this study, a panoramic radiograph for orthodontic purposes showed radiolucent areas in the maxilla and mandible, suggesting the presence of KCOTs. Chest radiography indicated the presence of rib anomalies, post anterior of the skull, computed tomography scan of the head and neck, which showed calcification of the cerebral falx and spina bifida which, according to Amezaga et al. [8], are characteristic of the syndrome.

KCOTs are among the most consistent and common features of NBCCS. They are found in 65 to 100% of affected individuals [14]. Clinically, the lesions are characterized by aggressive growth and a tendency to recur after surgical treatment. The epithelial cells of the basal layer show increased mitotic activity, together with a potential for budding and the presence of daughter cysts in the wall [14,15]. It has been reported that the presence of daughter cysts [16] was related to the recurrence of KCOT. The mandible is involved more frequently than the maxilla and the posterior regions are the most commonly affected sites [17].

There are two methods for the treatment of KCOT, a conservative and an aggressive. In the conservative method, simple enucleation with or without curettage and marsupialization are suggested. Aggressive methods include peripheral ostectomy, chemical curettage with Carnoy's solution, and resection [18].

Radical interventions as enucleation with shaving of surrounding bone or sometime resection might contribute to preventing recurrences and to improve the prognosis [16,18]. However serious consideration should be given to en bloc resection in the following cases: 1) when KCOT recurs despite previous enucleation with an adjunctive procedure; 2) when KCOT recurs despite previous marsupialization followed by enucleation with an adjunctive procedure; 3) in cases of multilocular (multilobular) aggressive intraosseous KCOT; 4) in cases of multiple nonsyndromic and syndromic KCOTs of NBCCS; or 5) in a diagnosed KCOT exhibiting particularly aggressive clinical behavior (eg, growth, destruction of adjacent tissues) that should require resection as the initial surgical treatment [19].

If the patient is in the first decade and has still unerupted permanent teeth involving KCOTs, it would be difficult to make a decision of aggressive surgery over conservative management. In children who have yet to be erupted, conservative management should be considered first because an aggressive operation can have an adverse effect on teeth development, the eruption process, and the development of the involved jaw [20]. Thus, younger patients usually receive more conservative than aggressive treatment [20,21].

Although some authors believe that simple enucleation might be the most appropriate conservative method for the treatment of KCOT [19,22], others have shown the successful treatment of large or multiple KCOTs using the marsupialization followed by enucleation [23-27]. Furthermore, it has been reported that marsupialization followed by enucleation results in the lowest recurrence rate among the conservative treatment [21,28]. Moreover, considering the complication of radical surgery, marsupialization followed by enucleation has been suggested as the conservative option for treatment of KCOT in younger patients [20,21,28].

Histopathological examination of the removed tumors should be performed to provide definitive diagnosis [8]. In this case, the microscopic analysis confirmed the diagnosis of KCOT and indicated the need for monitoring the disease. Long follow-up periods are suggested for this tumor [17]. In order to minimize secondary morbidities after the treatment, patients with KCOT should be observed carefully by radiographic imaging particularly during the first year [16].

This case reinforces the idea that the dentist, specially the pediatric and orthodontic specialties, has an important responsibility in early diagnosis and referral to other specialists for further evaluation. A definitive diagnosis of NBCCS should be made by a multidisciplinary team comprising medical specialists and dentists. Life expectancy in NBCCS is not significantly altered but there can be substantial morbidity as a result of complications [8]. Regular follow-up by a multi-specialists team should be offered. Moreover, early diagnosis is important for counseling of patients to prevent harmful exposure to ultraviolet and ionizing radiations that increase the risk of developing basal cell carcinoma [1,11]. The patient in this case study was sent to dermatologist for monitoring of possible skin lesions.

In summary, it can be said that Gorlin-Goltz syndrome is a dominant autosomal genetic process, which is of particular interest to the oral and maxillofacial health experts. Proper evaluation and characterization of the clinical features are of the utmost importance for the correct diagnosis and treatment of affected patients. In order to be able to establish early diagnosis of NBCCS, specialists should carry out clinical and imaging examinations in early ages of life. Physicians and dentists must know the features of the syndrome well.

## Competing interests

The authors declare that they have no competing interests.

## Authors' contributions

CAR and LDCNR drafted the manuscript. LVS and VVC carried out the histological analysis, wrote the histological part of the paper and contributed to the writing of the final version. ME, TCL and GVD analysed the patient's history, reviewed the patient data and surgically removed the tumors. Each author reviewed the paper for content and contributed to the writing of the manuscript. All authors approved the final report.
